# Focal breast edema and breast edema score on T2-weighted images provides valuable biological information for invasive breast cancer

**DOI:** 10.1186/s13244-023-01424-7

**Published:** 2023-04-30

**Authors:** Yanhong Chen, Lijun Wang, Ran Luo, Huanhuan Liu, Yuzhen Zhang, Dengbin Wang

**Affiliations:** Department of Radiology, Xinhua Hospital, Shanghai Jiao Tong University School of Medicine, No. 1665 Kongjiang Road, Shanghai, 200092 China

**Keywords:** Breast cancer, Edema, Breast edema score, Magnetic resonance imaging, Axillary lymph node metastasis

## Abstract

**Background:**

Various features extracted from breast MRI have the potential to serve as noninvasive biomarkers for the prediction of the biologic behavior of breast cancer. The purpose of this study was to investigate the value of focal breast edema and breast edema score (BES) on T2-weighted images in providing valuable biological information for breast cancer patients’ personalized treatment.

**Method:**

Two hundred and five lesions in 201 patients with invasive breast cancer confirmed by surgery or biopsy in Xinhua Hospital Affiliated to Shanghai Jiaotong University School of Medicine from November 2018 to October 2019 were retrospectively recruited and analyzed in this study. Focal edema and BES were evaluated at fat-suppressed T2 weighted imaging. All the lesions were divided into two groups according to the presence of focal edema. The differences in clinicopathological characteristics between the two groups and between different BES were compared.

**Results:**

Two hundred and five lesions in 201 patients with invasive breast cancer were retrospectively recruited and analyzed in this study. On the fat-suppressed T2WI, focal edema was detected in 102 of 205 lesions (49.8%). BES was positively correlated with tumor size (*p* < 0.001), histologic grade (*p* = 0.006), Ki-67 index (*p* < 0.001), and *N* stage (*p* = 0.007), and was negatively correlated with expression of ER and PR (*p* < 0.001). Higher BES was more likely to present in patients with non-luminal breast cancer (*p* < 0.001) and suggested the possibility of a higher *N* stage.

**Conclusions:**

Focal edema on T2WI of breast MRI indicates stronger tumor invasiveness, in which non-luminal breast cancer is more inclined to present focal edema. Breast edema score, a novel and practical tool, helps guide the individualized treatment of patients with invasive breast cancer.

**Graphical Abstract:**

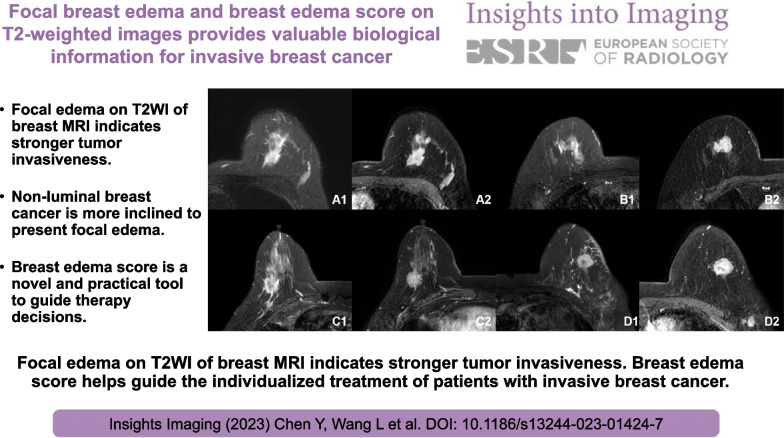

**Critical relevance statement:**

Focal edema on T2WI of breast MRI indicates stronger tumor invasiveness. Breast edema score helps guide the individualized treatment of patients with invasive breast cancer.

**Supplementary Information:**

The online version contains supplementary material available at 10.1186/s13244-023-01424-7.

## Key points

- Focal edema on T2WI of breast MRI indicates stronger tumor invasiveness.
- Non-luminal breast cancer is more inclined to present focal edema.
- Breast edema score is a novel and practical tool to guide therapy decisions.

**Supplementary Information:**

The online version contains supplementary material available at 10.1186/s13244-023-01424-7.

## Background

At present, breast cancer has become the most common malignancy in women. The latest data from the World Health Organization's International Agency for Research on Cancer showed that in 2020, there were 2.26 million new cases of breast cancer worldwide, with 685,000 deaths, ranking first in the world in terms of cancer incidence and female cancer deaths [1]. Breast magnetic resonance imaging (MRI) has been considered the most accurate imaging tool for providing anatomic information with multiple indications, varying from screening of high-risk women to breast cancer staging and neo-adjuvant chemotherapy (NACT) response evaluation [2].

Various features extracted from breast MRI have the potential to serve as noninvasive biomarkers that could predict the biologic behavior of the cancer. In current research, the dynamic contrast-enhanced magnetic resonance imaging (DCE-MRI) and diffusion weighted imaging (DWI) techniques are hotspots. By contrast, the research on the T2 fat suppression sequence has been less extensive. Previous studies have proved that T2-weighted imaging increases the specificity in differential diagnosis between benign and malignant lesions [3, 4]. Moreover, T2-weighted sequences allow the detection of edema in breast, which is more often associated with malignant lesions and is a biomarker of the aggressiveness of breast cancer [5, 6]. In 2015, Takayoshi Uematsu first comprehensively introduced focal and diffuse breast edema observed on the T2WI [6]. Breast focal edema were classified into three different types: peritumoral edema, prepectoral edema, and subcutaneous edema. Furthermore, Uematsu et al. firstly used breast edema score (BES) for classification of breast edema findings on T2-weighted MRI scans in 2021 [7].

However, a complete understanding of the potential clinical importance and prognostic value of focal edema has not yet been established. And to our knowledge, there are no previous published studies elaborately assessing the associations between breast focal edema on MRI and clinicopathological factors of breast cancer. Therefore, the purpose of this study was to investigate the correlation between focal edema on preoperative breast MRI and baseline clinicopathological characteristics in patients with mass-type invasive breast cancer, and further utilize BES to provide valuable biological information for patients’ personalized treatment.

## Methods

This retrospective study was approved by our Institutional Review Board and Ethical Committee, and the requirement for informed consent was waived.

### Study population

By reviewing our preoperative breast MR imaging database, we identified 319 malignant lesions confirmed by pathology between November 2018 and October 2019. Inclusion criteria were as follows: (1) pathological results were invasive breast cancer; (2) patients underwent breast MRI examination before operation; (3) complete clinical information; and (4) patients underwent axillary lymph node dissection or sentinel lymph node biopsy to confirm the status of axillary lymph nodes. Exclusion criteria were as follows: (a) the patients who had undergone any therapy before MRI examination and/or (b) breast MRI studies did not exhibit an enhancing lesion or only exhibit a non-mass enhancement, and/or (c) with insufficient image quality.

Finally, 201 patients with 205 lesions were included in this study. An overview of the patient selection process is shown in the Supplementary Material.

### MR imaging techniques

MR Imaging was performed on a 3.0-T MRI scanner (Ingenia, Philips, the Netherlands). The patients were positioned in the prone position with both breasts placed in an eight-channel phase-array breast coil. Firstly, axial T2-weighted imaging was obtained (TR/TE, 5000/65 ms; slice thickness/gap, 4/1 mm; flip angle, 90°; FOV, 37.2 cm; and matrix, 465 × 381). Next, axial diffusion-weighted imaging (DWI) (*b* = 0,800 s/mm^2^; TR/TE, 5100/72 ms; slice thickness/gap, 4/1 mm; flip angle, 90°; FOV, 35 cm; and matrix,136 × 140). Then, the dynamic imaging with enhanced T1 high resolution isotropic volume excitation (e_THRIVE) sequence (TR/TE, 4.2/2.1 ms; slice thickness/gap, 1/0 mm; flip angle, 12°; FOV 34 cm; matrix 407 × 404; and resolution 0.835 mm × 0.841 mm) were obtained before and four times immediately after the intravenous injection of Gadopentetate Dimeglumine (Gd-DTPA; Beilu, Beijing, China) with 0.1 mmol/kg as a bolus at a flow rate of 2 mL/s and 20 mL normal saline flush. A meantime of 6 s (range 4–8 s) was needed for the injection of contrast agent according to the weight of the patients. For each phase, the acquisition time was 65 s and the center of k-space acquisition time was 52 s. Contrast enhanced images were acquired at about 46, 111, 176, and 241 s after contrast material injection.

### MR imaging interpretation

MR images were analyzed independently by two experienced breast radiologists blinded to the clinical-pathological information (including the original MRI interpretation). In patients with multicentric/multifocal tumors, only the largest lesion was taken into account. The maximal diameter of the identified mass lesions was measured on DCE-MRI.

**Fig. 1:**
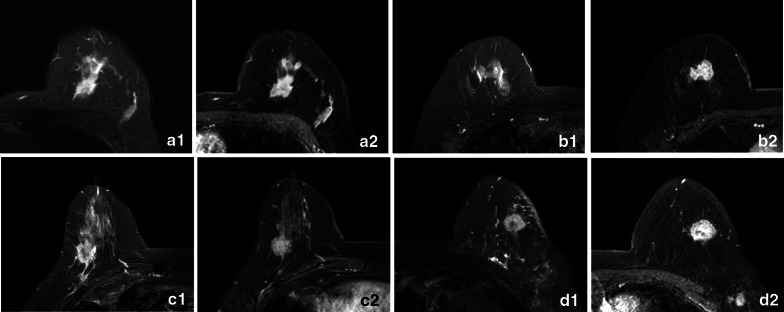
Examples of breast edema score on MRI. **a** A 43-year-old female presented with pathologically confirmed left breast cancer without axillary lymph node metastasis. IHC: ER (80% +), PR (90% +), KI67 (10% +), HER-2 (−). Axial fat-suppressed T2 weighted image (T2WI) showed no high signal intensity (no edema, BES 1) in the breast. Axial post-contrast T1 weighted image (DCE-MRI) showed the spiculated mass heterogeneously enhanced. **b** A 68-year-old female presented with pathologically confirmed right breast cancer with eight axillary lymph nodes metastasis. IHC: ER (−), PR (−), Ki-67 (30% +), HER-2 (−). T2WI showed the signal intensity around the tumor mass was as high as that of water (peritumoral edema, BES 2). DCE-MRI showed the apparent enhancement of lesion. **c** A 69-year-old female presented with pathologically confirmed right breast cancer with four axillary lymph nodes metastasis and lymphovascular invasion. IHC: ER (90% +), PR (70% +), KI67 (30% +), HER-2 (−). T2WI showed a line-shaped hyperintensity extended to the anterior space of pectoralis major muscle (prepectoral edema, BES 3) and high intensity around the mass. DCE-MRI showed the obvious enhancement of lesion. **d** A 64-year-old female presented with pathologically confirmed left breast cancer with fourteen axillary lymph nodes metastasis. IHC: ER (−), PR (−), KI67 (30% +), HER-2 ( +). T2WI showed the skin adjacent to the mass thickening and the hyperintensity of skin and subcutaneous fat (subcutaneous edema, BES 4) and high intensity in the prepectoral area. DCE-MRI showed the oval mass heterogeneously enhanced

Focal edema was assessed visually on pre-contrast fat-suppressed T2-weighted images. Focal edema, defined as high signal intensity (SI) as that of water on T2WI, were considered present around the tumor mass, or in the prepectoral region, or in the subcutaneous area. In accordance with previous articles about breast edema [7, 8], we used the breast edema score (BES) to classify findings of breast edema on T2-weighted images, as follows: BES 1, no edema (Fig. 1a); BES 2, peritumoral edema (Fig. 1b); BES 3, prepectoral edema (Fig. 1c); and BES 4, subcutaneous edema (Fig. 1d).

### Histopathological analysis

Histopathologic data, including tumor type, tumor grade, LN status, estrogen receptor (ER) status, progesterone receptor (PR) status, human epidermal growth factor receptor (HER2) status, Ki-67 index, and lymphovascular invasion (LVI) were obtained from the histopathologic reports after surgery or biopsy.

### Statistical analysis

All statistical analyses were performed using SPSS software, version 25.0 (IBM Corp.) and *p* < 0.05 was considered to indicate a statistically significant difference. All calculations were performed on a per-lesion basis. Comparisons of clinical-pathologic features between lesions with and without focal edema were performed. Intergroup comparison was performed using the Student’s t-test for independent variables with normal distribution and the Mann–Whitney U-test for variables with non-normal distribution. Comparison of categorical variables was performed using Pearson × 2 test or Fisher exact test. The correlation between different BES and clinical-pathological variables was examined by Kendall’s tau-b correlation coefficient.

Interobserver variability was calculated to evaluate the categorical ratings by two reviewers with k statics. The strength of k agreement was defined as follows: poor (*k* < 0), slight (*k* = 0.0–0.20), fair (*k* = 0.21–0.40), moderate (*k* = 0.41–0.60), substantial (k = 0.61–0.80), and almost perfect (*k* = 0.81–1.00).

## Results

### Patient characteristics and pathological classification

Totally, 205 lesions in 201 patients were included in this study. Four patients had synchronous bilateral invasive breast cancer. The mean age at the initial diagnosis was 57.9 ± 11.8 years, ranging from 29 to 88 years. One patient is male, and the rest are female. Median lesion size on MRI was 2.3 cm (range 0.6–6.9 cm).

Pathological results were obtained by surgery or biopsy in all 205 lesions (Table 1). There was no significant difference in pathological classification between lesions with focal edema and those without focal edema (*p* > 0.05).

**Table 1:** Correlation between focal edema and histopathological type of breast cancer

Histopathological type	Focal edema	No focal edema	*p* value
Invasive ductal carcinoma (IDC)	94	86	0.078
Invasive lobular carcinoma (ILC)	2	3	
Invasive papillary carcinoma	3	4	
Invasive mucinous carcinoma	1	9	
Apocrine carcinoma	2	1	
Total	102	103	

### Presence of edema

On the T2WI with fat suppression, none in the 205 lesions exhibited diffuse edema, 102 lesions (49.8%) exhibited focal edema and the remaining 103 lesions (50.2%) did not exhibit edema. In the 102 lesions with focal edema, thirteen lesions displayed both peritumoral edema and prepectoral edema, while the other 12 lesions displayed both peritumoral edema and subcutaneous edema, and another 10 lesions displayed all three types of focal edema. Conclusively, of all 205 lesions, 103 had BES 1, 55 had BES 2, 31 had BES 3, and 16 had BES 4.

### Focal edema

Table 2 describes the correlation between focal edema and clinical-pathological features in invasive breast cancer. Focal edema was significantly more common in larger tumors. The incidence of focal edema in lesions larger than 2 cm (68.8%) had twice as that in smaller lesions (≤ 2 cm) (33.0%) (*p* < 0.001). Focal edema was more frequently found in patients with higher histologic grade (*p* = 0.010), negative ER status (*p* < 0.001), negative PR status (*p* = 0.002), higher Ki-67 index (*p* < 0.001), and positive HER2 status (*p* = 0.034). Patients with non-luminal breast cancer (HER2-positive and triple-negative) were more inclined to present focal edema than those with luminal breast cancer with a statistically highly significant *p*-value (*p* < 0.001) (Fig. 1b and d). A higher rate of axillary lymph node metastasis was found in patients with focal edema (*p* = 0.031).

**Table 2:** Correlation between focal edema and clinical-pathological features in invasive breast cancer

	Focal edema^†^		*p* value
	−(*n* = 103)	+(*n* = 102)	
Age in years, mean ± SD	57.04 ± 12.01	58.69 ± 11.70	0.321
Tumor size on MRI in cm (IQR)	1.7 (1.3~2.2)	2.4 (1.9~3.2)	** < 0.001**
*cT stage*			
T1	73	36	** < 0.001**
T2-3	30	66	
Grade*			
WHO I/II	54	39	**0.010**
III	26	43	
*ER*			
Status in percentage (IQR)	90 (75~90)	70 (0~90)	** < 0.001**
Negative	11	33	** < 0.001**
Positive	92	69	
*PR*			
Status in percentage (IQR)	70 (20~90)	10 (0~70)	** < 0.001**
Negative	18	37	**0.002**
Positive	85	65	
*Ki-67*			
Status in percentage (IQR)	18 (10~30)	30 (15~40)	** < 0.001**
Low, < 14%	42	17	** < 0.001**
High, ≥ 14%	61	85	
*HER-2*			
Negative	92	80	**0.034**
Positive	11	22	
*LN status*			
Negative	78	63	**0.031**
Positive	25	39	
pN stage**			
N0	78	63	0.114, N0 versus N1 0.814
N1	19	14	N0 versus N2-3 **0.011**
N2-3	5	15	N1 versus N2-3 **0.021**
*LVI*			
Negative	90	98	0.073
Positive	12	5	
*Molecular subtype*			
Luminal	93	71	** < 0.001**
Non-luminal	10	31	

Statistically signifcant *p*-values are bolded

^†^As long as one of peritumoral edema, prepectoral edema and subcutaneous edema occurs in the breast it is considered as positive for focal edema

*Data from 43 patients were unavailable

**Data from 11 patients were unavailable. They only underwent sentinel lymph node biopsy without axillary lymph node dissection or underwent axillary lymph node dissection after neoadjuvant chemotherapy

### Breast edema score

Table 3 shows the correlation between breast edema score and clinical-pathological features in invasive breast cancer. For correlation analysis with Kendall’s tau-b correlation coefficient, BES was positively correlated with tumor size (*p* < 0.001), histologic grade (*p* = 0.006), Ki-67 index (*p* < 0.001), and N stage (*p* = 0.007), and was negatively correlated with expression of ER and PR (*p* < 0.001). Lesions with higher BES were more prone to be HER-2 positive (*p* = 0.007) and the molecular subtype was more likely to be non-luminal (*p* < 0.001).

**Table 3:** Clinical-pathological features of invasive breast cancer with different breast edema score

	BES1	BES2	BES3	BES4	*p* value	Intergroup comparison					
	(*n* = 103)	(*n* = 55)	(*n* = 31)	(*n* = 16)		BES 1 versus 2	BES 1 versus 3	BES 1 versus 4	BES 2 versus 3	BES 2 versus 4	BES 3 versus 4
Age in years	57.0 ± 12.0	58.5 ± 12.1	56.0 ± 11.3	64.6 ± 9.2	0.256	0.483	0.679	**0.017**	0.365	0.064	**0.012**
Tumor size on MRI in cm	1.9 ± 0.9	2.3 ± 0.8	3.3 ± 1.4	2.5 ± 0.9	** < 0.001**	0.483	** < 0.001**	**0.007**	** < 0.001**	0.216	0.058
*cT stage*											
T1	73	25	4	7	** < 0.001**	**0.002**	** < 0.001**	**0.032**	**0.002**	0.904	**0.045**
T2–3	30	30	27	9							
Grade*											
WHO I/II	54	24	9	6	**0.006**	0.117	**0.014**	0.077	0.268	0.493	0.823
III	26	21	14	8							
*ER*											
Status in percentage	74.4 ± 31.3	46.6 ± 45.7	39.3 ± 41.7	46.6 ± 45.7	** < 0.001**	0.061	** < 0.001**	0.075	**0.019**	0.222	0.581
Negative	11	14	13	6	** < 0.001**	**0.015**	** < 0.001**	**0.014**	0.114	0.531	0.769
Positive	92	41	18	10							
*PR*											
Status in percentage	54.1 ± 35.4	38.9 ± 37.6	18.35 ± 28.5	24.4 ± 31.6	** < 0.001**	**0.013**	** < 0.001**	**0.002**	**0.013**	0.215	0.511
Negative	18	16	15	6	**0.001**	0.091	** < 0.001**	0.128	0.074	0.739	0.477
Positive	85	39	16	10							
*Ki-67*											
Status in percentage	21.6 ± 17.6	29.1 ± 20.5	43.5 ± 23.1	27.3 ± 17.8	** < 0.001**	**0.018**	** < 0.001**	0.230	**0.004**	0.757	**0.018**
Low < 14%	51	19	3	5	** < 0.001**	0.071	** < 0.001**	0.173	**0.011**	0.806	0.146
High ≥ 14%	52	36	28	11							
*HER-2*											
Negative	92	47	22	11	**0.007**	0.477	**0.026**	0.064	0.105	0.249	1.000
Positive	11	8	9	5							
*LN status*											
Negative	78	39	15	9	0.074	0.510	**0.004**	0.183	**0.038**	0.270	0.609
Positive	25	16	16	7							
*pN stage*											
N0	78	39	15	9	**0.007**						
N1	19	8	4	2							
N2–3	5	6	6	3							
*LVI*											
Negative	98	49	27	14	0.055	0.273	0.246	0.238	1.000	1.000	1.000
Positive	5	6	4	2							
*Molecular subtype*											
Luminal	10	13	13	5	** < 0.001**	**0.018**	** < 0.001**	**0.044**	0.076	0.772	0.475
Non-luminal	93	42	18	11							

Statistically signifcant *p*-values are bolded

*Data from 43 patients were unavailable

**Fig. 2:**
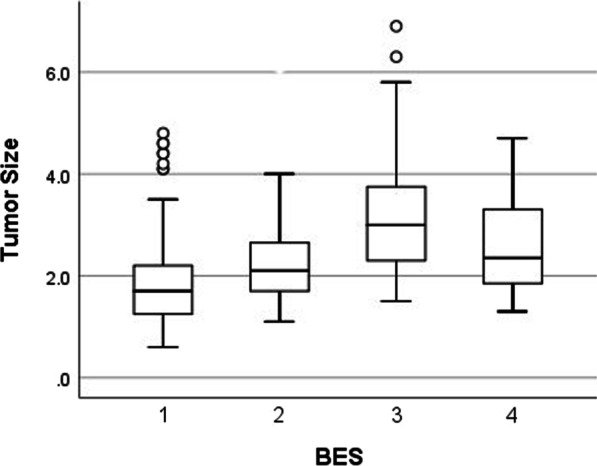
Tumor size in different breast edema score

For intergroup comparison, lesions with BES 3 compared with BES 1 and BES 2 exhibited larger tumor size, lower expression of ER/PR, higher Ki-67 index and higher prevalence of axillary lymph node involvement. BES 1 and 2 did not differ significantly in axillary lymph node metastasis. Excluding BES 4, larger tumors appeared in higher breast edema scores (Fig. 2). A trend was observed in BES; higher N stage was seen in higher BES. Patients in N2-3 accounted for 24% and 22% in the BES3 and 4 groups, much higher than in the BES1 and 2 groups (5%, 11%).

### Interobserver agreement

With regard to the interobserver agreement between the two radiologists, an almost perfect agreement was observed for focal edema (*k* = 0.844) and substantial agreement for BES (*k* = 0.691).

## Discussion

The purpose of this study was to clarify the association between focal edema and tumor stage, immunohistochemical results, and axillary lymph node metastasis to determine the clinical prognostic value of T2 fat-suppressed sequences in breast cancer. Our results showed that the presence of focal edema and breast edema score were closely associated with the characteristics of biologically aggressive tumors (larger tumor size, lower expression of ER/PR, higher Ki-67 index, positive HER2 status and lymph node metastasis). Lesions with higher BES were more possible to present a higher N stage and to be non-luminal subtype, indicating that breast edema score could predict worse prognosis and probably contribute to disease recurrence.

Lymph node metastasis, an important prognostic factor, is an influencing factor to the patient's treatment strategy [9]. Most previous studies have focused on the relationship between axillary lymph node metastasis and clinicopathological features or imaging features on DCE-MRI or DWI [10, 11]. This study analyzed the correlation between breast edema and axillary lymph node metastasis, which is rarely seen in previous studies [8]. Breast focal edema can be assessed by conventional T2WI without contrast agents, which has high clinical application value and needs to be further explored. This study proved that breast focal edema was closely related to axillary lymph node metastasis, and higher BES suggested the possibility of a higher N stage.

In our study, lesions with BES 3 exhibited significant differences with BES 1 and BES 2 in some prognostic factors, such as larger tumor size, lower expression of ER/PR, higher Ki-67 index and higher prevalence of axillary lymph node involvement. Previous studies proposed that patients with breast cancer with prepectoral edema (BES 3) are at an early stage of occult inflammatory breast cancer (IBC) [7]. When the dilated lymphovascular system is filled with cancer cells in the retromammary area, prepectoral edema appears. Lymphovascular invasion (LVI) is the pathological finding of tumor emboli present within both lymphatic and vascular systems at the periphery of an invasive breast cancer [12], which has been reported to have a prognostic significance [13] and is valuable for predicting the pathological response and outcome of patients who receive NAC [14]. Previous studies [6, 15] reported that peritumoral edema (BES 2), prepectoral edema (BES 3), and subcutaneous edema (BES 4) are all associated with the presence of LVI, which was related to the tumor microenvironment. But in our study, due to less detection of LVI, lymphovascular invasion showed no association with breast edema.

These results further confirmed that focal edema detected on T2-weighted images can be considered a valid additional tool to obtain further information about breast cancer biology, contributing to precision medicine [4, 7, 16–19]. It is relatively more feasible for clinicians or junior breast radiologists to identify focal edema. As for senior breast radiologists, they could use BES to obtain more information from the imaging. As a potential imaging biomarker for the comprehensive risk classification and prognostication for patients with breast cancer, BES may be valuable for defining the appropriate therapeutic targets for patients who need a more intensive adjuvant management. Women with a higher risk of early relapse appear to benefit from the use of novel or more intensive adjuvant treatment. In addition, the assessment of focal edema is practical because its presence can be readily evaluated by using conventional T2-weighted images without contrast agents with a great interobserver agreement.

The present study has some limitations. First, it is a retrospective analysis. We plan to enrich our results with a long-term evaluation of recurrence-free survival and distant metastasis-free survival afterward. Second, we did not include non-mass enhancements, most of which were confirmed to be ductal carcinoma in situ (DCIS), and per definition, these lesions have interspersed areas of fatty tissue and normal fibroglandular, which could affect the measurements. Third, the assessment of focal edema on breast MRI was subjective despite great agreement between observers. In the future, we will further apply radiomics and deep learning methods to confirm the utility of MRI imaging biomarkers such as breast edema in clinical practice.

## Conclusions

This study demonstrated that focal breast edema of breast cancer observed on preoperative MRI indicates stronger tumor invasiveness. Breast carcinomas with focal edema showed a higher prevalence of axillary lymph node involvement, with more HER 2 + , lower prevalence of ER +/PR + , and higher Ki-67 index, in which the non-luminal subtype accounted for the majority. For this reason, the evaluation of breast edema score on T2WI, a novel and practical tool, could optimize personalized treatment for breast cancer patients.

## Supplementary Information

**Additional file 1.** Flowchart of study enrollment.

## Data Availability

The datasets generated and/or analyzed during the current study are not publicly available but are available from the corresponding author upon a reasonable request.

## Abbreviations

BES: Breast edema score
DCE-MRI: Dynamic contrast-enhanced magnetic resonance imaging
DCIS: Ductal carcinoma in situ
DWI: Diffusion weighted imaging
ER: Estrogen receptor
HER2: Human epidermal growth factor receptor 2
IBC: Inflammatory breast cancer
LVI: Lymphovascular invasion
MRI: Magnetic resonance imaging
NACT: Neo-adjuvant chemotherapy
PR: Progesterone receptor
SI: Signal intensity
