# Supplementary material for: Focal breast edema and breast edema score on T2-weighted images provides valuable biological information for invasive breast cancer
Source: Insights Imaging. 2023 Apr 30;14:73. doi: 10.1186/s13244-023-01424-7 (PMC10149534; doi:10.1186/s13244-023-01424-7)
Supplement: Supplementary file 1 — Additional file 1. Flowchart of study enrollment. [file 13244_2023_1424_MOESM1_ESM.pdf]

ELECTRONIC SUPPLEMENTARY MATERIAL

Focal breast edema and breast edema score on T2-weighted images provides valuable biological information for invasive breast cancer

Flowchart of the study enrollment

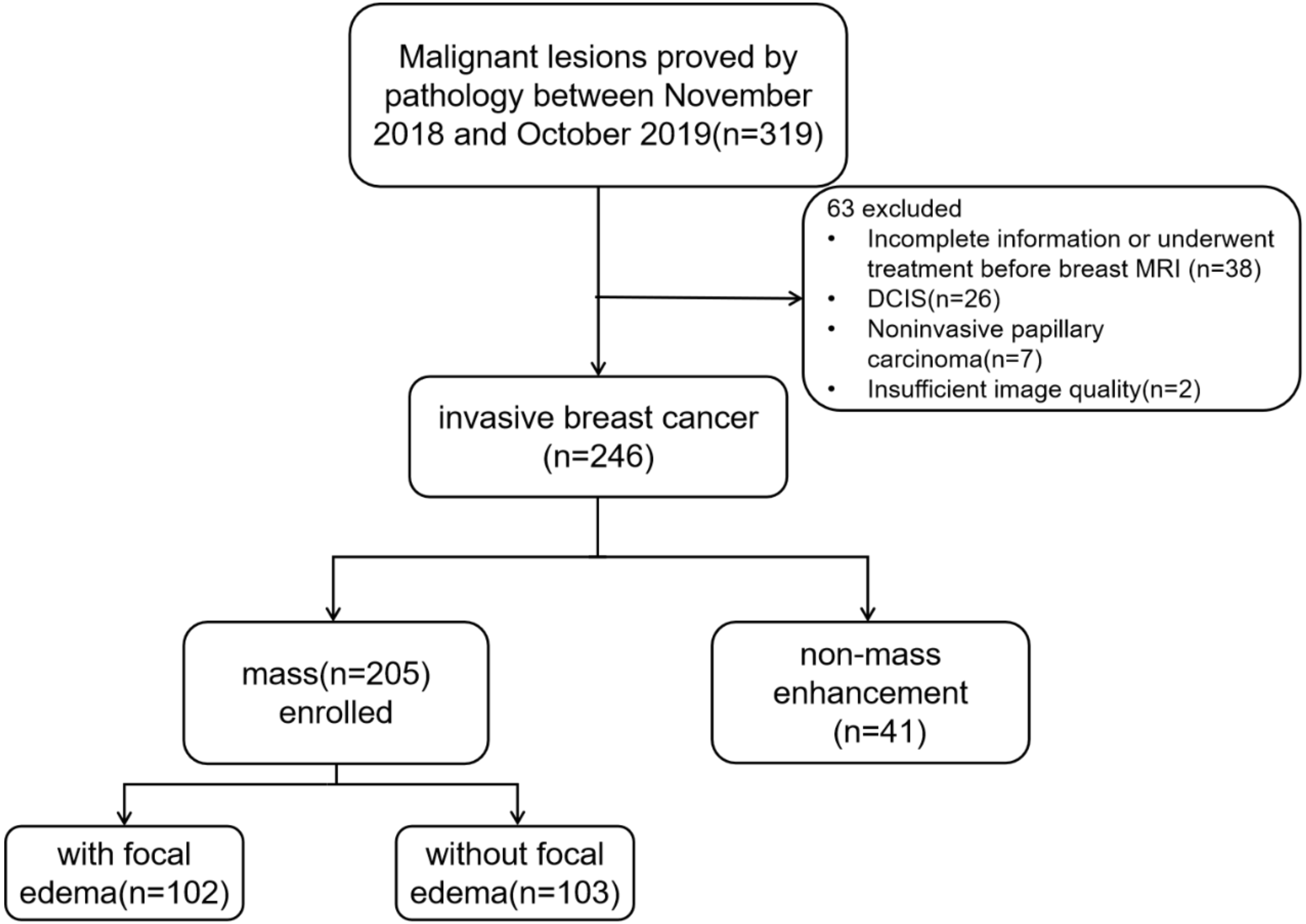

Insights Imaging (2023) Chen Y, Wang L, Luo R, Liu H, Zhang Y, Wang D
